# The Adult Patient with Eisenmenger Syndrome: A Medical Update after Dana Point Part III: Specific Management and Surgical Aspects

**DOI:** 10.2174/157340310793566127

**Published:** 2010-11

**Authors:** Erwin Oechslin, Siegrun Mebus, Ingram Schulze-Neick, Koichiro Niwa, Pedro T Trindade, Andreas Eicken, Alfred Hager, Irene Lang, John Hess, Harald Kaemmerer

**Affiliations:** 1 Congenital Cardiac Centre for Adults, University Health Network/Toronto General Hospital/Peter Munk Cardiac Centre, 585 University Avenue, Toronto, ON. M5G 2N2, Canada; 2 Department of Pediatric Cardiology and Congenital Heart Disease, Deutsches Herzzentrum München, Technische Universität München, München, Germany; 3 UK Service for Pulmonary Hypertension in Children, Cardiac Unit, Great Ormond Street Hospital, London, WC1N 3JH, United Kingdom; 4 Department of Pediatrics, Chiba Cardiovascular Center, 575 Tsurumai, Ichihara, Chiba 290-0512, Japan; 5 Department of Cardiology, University Hospital Zurich, Rämistrasse 100, 8091 Zürich, Switzerland; 6 Department of Cardiology, University of Vienna, Währinger Gürtel 18-20, 1090 Wien, Austria

**Keywords:** Cardiovascular diseases, adult congenital heart defects, pulmonary hypertension, Eisenmenger syndrome, follow-up studies, Competence Network for Congenital Heart Defects.

## Abstract

Eisenmenger syndrome is the most severe form of pulmonary arterial hypertension and arises on the basis of congenital heart disease with a systemic-to-pulmonary shunt. Due to the chronic slow progressive hypoxemia with central cyanosis, adult patients with the Eisenmenger syndrome suffer from a complex and multisystemic disorder including coagulation disorders (bleeding complications and paradoxical embolisms), renal dysfunction, hypertrophic osteoarthropathy, heart failure, reduced quality of life and premature death.

For a long time, therapy has been limited to symptomatic options or lung or combined heart-lung transplantation. As new selective pulmonary vasodilators have become available and proven to be beneficial in various forms of pulmonary arterial hypertension, this targeted medical treatment has been expected to show promising effects with a delay of deterioration also in Eisenmenger patients. Unfortunately, data in Eisenmenger patients suffer from small patient numbers and a lack of randomized controlled studies.

To optimize the quality of life and the outcome, referral of Eisenmenger patients to spezialized centers is required. In such centers, specific interdisciplinary management strategies of physicians specialized on congenital heart diseases and PAH should be warranted.

This medical update emphasizes the current diagnostic and therapeutic options for Eisenmenger patients with particularly focussing on specific management and surgical aspects.

## SPECIFIC MANAGEMENT ASPECTS

### 1.1 Erythrocytosis - Phlebotomy

Secondary **erythrocytosis** due to increased erythropoietin production is a physiologic, adaptive response to chronic hypoxemia and is the appropriate term to describe the increased mass of red blood cells. Polycythemia is a completely inappropriate term as it describes a malignant clonal stem cell disorder of all three cell lines - including red blood cells, white blood cell and platelets. This is in contrast to patients with Eisenmenger syndrome (ES) who have even a decreased amount of platelets. 

There is an inverse relationship between cyanosis (oxygen saturation) and red blood cell count: the lower the oxygen saturation, the higher the red blood cell count and the hematocrit in iron replete patients [1]. There is a direct relationship between red blood cell mass and exercise capacity despite higher blood viscosity [2]. 

Blood viscosity depends on many factors: red blood cell mass and morphology, aggregation and dispersion of blood cells, plasma viscosity, temperature and shear stress [2-4]. Secondary erythrocytosis results in increased shear stress, which modifies the balance between vasodilators and vasoconstrictors. Cyanosis and secondary erythrocytosis affect the pulmonary and systemic vascular system. Severe systemic endothelial dysfunction is evident in patients with cyanotic CHD as evidenced by the striking impairment of endothelium-dependent vasodilation induced by acetylcholine [5]. It is not clear whether these findings are clinically relevant, but it is conceivable that severe endothelial vasodilator dysfunction may be involved in the development of cardiovascular events in Eisenmenger patients [5]. 

Compensated secondary erythrocytosis (stable hemoglobin in an iron-replete state) represents an appropriate, adaptive response to chronic hypoxemia. Hyperviscosity symptoms are absent, mild or moderate even at hematocrit levels higher than 70%. Patients with decompensated erythrocytosis have failed to establish an equilibrium and complain of moderate to severe hyperviscosity symptoms [6-9]. 

Systematic assessment of hyperviscosity symptoms at each clinical visit is essential and allows a precise clinical follow-up and early detection of clinical deterioration (Table **1**) [9]. 

There is an ongoing debate whether iron deficiency causes or contributes to symptoms, whole blood hyperviscosity and risk of stroke. Indeed, there are controversial data if iron deficiency impacts whole blood hyperviscosity [10-16]. A recent study showed that iron deficiency did not alter blood viscosity [2]. However, there are several limitations of such an *in vitro* study as simplified steady state models are used to measure infinitely more complex and dynamic systems *in vivo* and the data may not apply to *in vivo* conditions.

Iron deficiency may cause headache, reduced exercise tolerance, restless leg syndrome [17, 18]. Despite the discrepancy of data and ongoing controversy, iron deficiency must be avoided in patients with ES. A patient in an iron-deplete state cannot produce hemoglobin at a level adequate and appropriate to the severity of cyanosis (the lower the oxygen saturation, the higher the hemoglobin).

#### Indications for Phlebotomy

Prophylactic or routine phlebotomies to maintain the hematocrit level within an arbitrary level and for the prevention of cerebrovascular events are not justified [4, 8, 9, 19, 20]. Unfortunately, repeat, injudicious phlebotomies are still performed in daily practice. This practice must be discouraged because it causes iron deficiency, reduced exercise tolerance, impaired oxygen transport capacity due to anemia and increased risk of cerebrovascular events [1, 2, 21]. Hemoglobin levels that are physiologic for an acyanotic patient are misinterpreted to be adequate for cyanotic patients although relative iron-deficiency anemia is present. Relative iron deficiency anemia is often ignored or not recognized in cyanotic patients as the hemoglobin may be less than 15 g/dl but should be greater than 18 g/dl [9]. Severity of secondary erythrocytosis per se is not a risk factor for stroke [21, 22]. Thus, an increased hemoglobin level, in and of itself, is no indication for phlebotomies and poses the potential hazards of iron deficiency. The primary goal of phlebotomy is to relieve temporarily moderate to severe hyperviscosity symptoms. Therapeutic phlebotomy is restricted to patients with moderate to severe hyperviscosity symptoms in the absence of both volume depletion and iron deficiency (Table **2**). 

There are two indications for phlebotomies:

1. moderate to severe hyperviscosity symptoms due to secondary erythrocytosis;
2. preoperative phlebotomy for autologous blood donation if the hematocrit level is above 65%.

Hyperviscosity will disappear after adequate phlebotomy within 24 hours resulting in increased cardiac output and systemic blood flow due to decreased whole blood viscosity and decreased systemic vascular resistance [23, 24].

#### Phlebotomy Procedure

Phlebotomy can be a safe procedure, if the following safety measures are followed:

- Assessment of baseline vital signs and every 15 minutes for the 1^st^ hour after the procedure (more frequently if the patient is symptomatic);
- Insertion of bilateral intravenous lines allowing fluid replacement and phlebotomy;
- Some centers recommend air filters to avoid air embolism;
- Preceding or concurrent infusion of 750 to 1000 cc of isotonic saline (at least over 1 hour) while phlebotomizing 500 cc of blood;
- Stop the procedure if the patient experiences severe hypotension, symptomatic palpitations, or pre-syncope/ syncope.

Volume depletion or brain abscess must be strongly considered, if hyperviscosity symptoms (headache!) persist or do not improve after phlebotomy. A brain abscess may be initially missed and interpreted as hyperviscosity symptom. If hyperviscosity symptoms persist in the absence of volume depletion and abscess, a second phlebotomy performed within 48 hours may improve the symptoms.

### 1.2 Hemostatic Abnormalities / Thrombosis / Paradoxical Embolism

#### 1.2.1 Hemostatic Abnormalities

Hemostatic abnormalities are common and complex in cyanotic patients. They are attributed to abnormalities in platelets, coagulation pathways and other coagulation mechanisms. 

##### Platelets:

platelet abnormalities include both *thrombocytopenia* and *thrombasthenia*. There is a positive correlation between platelet count and oxygen saturation or an inverse relationship with hemoglobin/ hematocrit level. Platelet counts are usually in the lower range of normal or reduced due to decreased production because of ineffective thrombopoiesis [25]. In addition, platelet survival is reduced [26], and platelet function measured by whole blood impedance aggregometry is decreased in patients with elevated hematocrit.^1^

##### Abnormal coagulation parameters:

Vitamin K dependent clotting factors (factors II, VII, IX, X) and factor V are reduced. Increased fibrinolytic activity and depletion of the largest von Willebrand multimers contribute to the bleeding tendency in these patients [27, 28]. In this process, cyanosis, pulmonary vascular disease, and turbulent blood flow are major determinants of the von Willebrand abnormality, which appears to be acquired and may contribute to the bleeding diathesis [7]. Bleeding time fails to demonstrate hemostatic abnormalities as it is paradoxically shorter in cyanotic patients than in controls even though platelet counts/ function and coagulation parameters are abnormal. High blood viscosity resulting in impaired blood flow may explain this observation.

##### Vascular factors:

Surgical observations have suggested increased tissue vascularity in ES patients. Endothelial derived nitric oxide and other vasodilators are released by the increased shear stress and results in arteriolar dilatation. By contrast, blood flow in the forearm was lower in cyanotic patients than in healthy subjects [5].

#### 1.2.2 Bleeding and Thrombotic Diathesis

There is a paradox invoking a therapeutic dilemma: patients with ES are at risk for both bleeding and thrombosis. The coagulation abnormalities increase the risk of **spontaneous bleeding,** which is usually mild, self-limited and not life-threatening in Eisenmenger patients (Table **1**). The use of a toothbrush with soft bristles and gentle brushing help to avoid or minimize gingival bleeding.

**Hemoptysis** (which is discussed below) is the most common and life-threatening bleeding complication, the others are less common [29, 30]. A multidisciplinary team including coagulation experts and other specialists can best manage major hemorrhage and the risk of serious morbidity and mortality [8].

**Thrombosis** is facilitated by many factors: coagulation abnormalities, stasis of blood in the dilated flow chambers and vessels, atherosclerosis and/ or endothelial injury, the presence of thrombotic material (e.g. artificial valves, conduits), arrhythmias (atrial flutter/ atrial fibrillation). Interestingly, the hemostatic abnormalities do not protect against blood clot formation. Laminated thrombi in the large, partially calcified, and aneurysmal pulmonary arteries are common and occur in up to 30% of ES patients [29, 31-33], Fig. (**1**).

In situ thrombi can be the source of artery-to-artery intrapulmonary emboli resulting in pulmonary infarction and intrapulmonary hemorrhage. Female gender and low oxygen saturation were identified as risk factors for thrombus formation in the proximal pulmonary arteries [32]. This is in contrast to another study, where in situ thrombosis in pulmonary arteries were related to older age, biventricular dysfunction, poor functional class (NYHA class III and IV) and dilatation of pulmonary arteries and concomitant slow pulmonary blood flow. The degree of cyanosis or coagulation abnormalities did not differ between patients with and without thrombus formation in the central pulmonary arteries [33]. The occurrence or absence of thrombus does not seem to have an impact on survival [30]. 

The key question remains: can long-term anticoagulation prevent thrombus formation in the central pulmonary artery and is there any benefit or hazard to Eisenmenger patients? Thrombus formation and bleeding coexist in patients with ES. There are no clinical data (see also Part II “1.1 Anticoagulation”) to show effectiveness and benefit of routine anticoagulation or aspirin therapy in this population in the absence of any other strong indication (e.g. persistent atrial fibrillation/ flutter). Anticoagulation may be offered to patients with thrombus material in the central pulmonary artery or in case of pulmonary artery-to-pulmonary artery embolism. However, meticulous monitoring and adjustment of the amount of sodium citrate in the test tubes is needed if an Eisenmenger patient is on anticoagulants. Although anticoagulation has been shown to reduce morbidity and mortality in patients with idiopathic PAH it is tempting to draw similar conclusions for adults with ES. However, there are no data to support this approach and recommendations for routine anticoagulation cannot be given to patients with ES [20].

##### Risk Reduction Strategies

The risk of anticoagulation must be carefully assessed as both prophylactic and therapeutic anticoagulation reinforces hemostatic abnormalities [8].

- Strategies to reduce the risk of bleeding include:
  - Limitation of anticoagulation to urgent indications for anticoagulation such as atrial fibrillation, recurrent thromboembolic events, mechanical heart valve prostheses prostheses;
  - Meticulous surveillance of anticoagulation; the optimal range of the INR or aPTT has not been evaluated. Recommendations for therapeutic anticoagulation are a target INR between 2.0 and 2.5 (in the absence of a mechanical valve) or a therapeutic aPTT of 1.5 times of the normal value;
  - Prompt therapy of respiratory tract infections.
- Strategies to reduce the risk of ischemic events include:
  - Avoidance and treatment of volume depletion;
  - Iron supplementation in patients with iron deficiency or those undergoing repeated phlebotomies;
  - Use of air filters in all intravenous lines.

#### 1.2.3 Paradoxical Emboli

Patient with ES are at risk for paradoxical emboli as there is a usually wide communication between the pulmonary and systemic circulation. Intravenous pacemaker or ICD systems are contraindicated as transvenous leads are associated with a 2-fold increased risk of systemic thromboemboli in patients with intracardiac shunts [34]. 

**Risk reduction strategies** to reduce morbidity and mortality include:

- Relative contraindication of intravenous implantation of pacemaker or ICD;
- Air filters in all intravenous lines to prevent air embolism.

### 1.3 Cerebrovascular Events

Patients with ES have an increased risk of cerebrovascular events which is reported to occur in up to 14% of patients [21]. The causes of cerebrovascular events can be multifactorial: paradoxical emboli, rheological problems, e.g. microcytosis, and “traditional” risk factors, like hypertension and atrial fibrillation, are believed to contribute to this increased risk. Severe endothelial dysfunction may also be involved in the development of cardiovascular events in patients with cyanotic CHD as abnormal vascular regulatory mechanisms may contribute to the increased risk for ischemic complications; nitric oxide is not only a potent vasodilator but also a powerful antiaggregatory agent [5, 35].

The misconception of secondary erythrocytosis being a risk factor for cerebrovascular events has been clarified: the severity of secondary erythrocytosis *per se* is not a risk factor [21, 22, 30]. Microcytosis caused by iron deficiency, usually due to repeated, inappropriate phlebotomies, was the strongest independent predictor for cerebrovascular events [21]. 

#### Risk Reduction Strategy

- Avoidance and treatment of volume depletion;
- Air filters in all intravenous lines to prevent air embolism;
- Iron supplementation in patients being iron deficient or subjected to recurrent phlebotomy.

### 1.4 Renal Dysfunction

Renal dysfunction affects two thirds of adults with ES and has a strong impact on mortality in patients with at least moderate renal dysfunction (Glomerular Filtration Rate < 60 mL min^-1^ 1.73 m^-2^) [36]. Both functional and structural abnormalities of the kidneys occur in patients with cyanotic congenital disease and the underlying mechanisms leading renal impairment are multifactorial and complex and are still a matter of debate [4, 37]. Volume depletion and/ or administration of nonsteroidal anti-inflammatory drugs can be fatal. Thus, routine assessment of kidney function is recommended not only for those on nephrotoxic drugs, but for all Eisenmenger patients to obtain prognostic information (Table **3**).

### 1.5 Hemoptysis

This life-threatening complication is common in patients with ES and reported in up to 100% [30, 38, 39]. Rupture of aortopulmonary collaterals or of an aneurysm of the pulmonary artery is usually fatal, Fig. (**1**).

Fortunately, hemoptysis is not a common mode of death [38-40]. Hemoptysis is an *external manifestation of an intrapulmonary hemorrhage* and does not reflect the extent of bleeding. Major hemoptysis requires evaluation by a multidisciplinary team. 

**General diagnostic and therapeutic aspects** include [8]:

- Hospital admission;
- Reduction of physical activity and suppression of nonproductive cough;
- Chest x-ray followed by thoracic computed tomography if there are infiltrates on the chest x-ray, in order to assess the severity of intrapulmonary bleeding or to visualize *in situ* thromboses in the proximal dilated pulmonary arteries;
- Avoidance of bronchoscopy as it rarely provides useful information, but it confers risks. Bronchoscopy may be performed by an expert team including anesthetists to identify the location of bleeding if the location/ source of the bleeding is unclear and subsequent angiography and coil occlusion of the collateral vessels are considered;
- Immediate discontinuation of aspirin, nonsteroidal anti-inflammatory drugs and oral anticoagulants;
- Treatment of hypovolemia and anemia. Hemoglobin must be adequate to the degree of cyanosis!

**Specific diagnostic/ therapeutic aspects** may be needed, if hemoptysis is severe or incessant:

- Consider administration of platelets in the presence of low platelet counts and/ or administration of fresh frozen plasma, vitamin K or coagulation factors;
- Angiography with selective embolization of the artery supplying the source of blood loss;
- Sputum culture and treatment of infectious disease. Consider tuberculosis as a cause of hemoptysis.

Risk reduction strategy:

- Immediate treatment of respiratory tract infections;
- Pneumovax and annual flu shot to prevent respiratory tract and pulmonary infections.

### 1.6 Gout

Increased uric acid levels (hyperuricemia) are common in patients with cyanotic CHD and result from increased production and decreased renal clearance [41]. In adults, renal hypoperfusion reinforced by a high filtration fraction enhances urate reabsorption and secondary hyperuricemia [41]. In contrast to primary hyperuricemia, secondary hyperuricemia seems to have little or no deleterious effect on renal function as soft tissue deposits are the exception in cyanotic patients [42]. 

Serum uric acid increases in proportion to hemodynamic severity in adults with ES and is independently associated with long term mortality [43]. Routine assessment of serum uric acid is inexpensive and may serve as an indicator for disease severity during follow-up (Table **3**).

Clinical presentation of primary and secondary gout is similar, but acute gouty arthritis is less prevalent in adults with ES [41, 42]. 

Asymptomatic, secondary hyperuricemia is no indication for routine therapy to lower uric acid level because it does not have any serious impact on renal function. However, acute gouty arthritis, a very painful complication, responds well to oral or intravenous colchicine. Intravenous administration may be preferred during the acute phase to obviate the gastro-intestinal side effects (nausea, vomiting, diarrhea, volume depletion). This effective therapeutic measure may be continued with a low oral dose of colchicine to prevent recurrent episodes. Intraarticular administration of corticosteroids may be needed in selected patients (e.g. acute gouty arthritis of the knees).

Uricosuric agents (e.g. probenecid) or uricostatic agents (e.g. allopurinol) are treatment options in patients with recurrent gouty arthritis. Gouty arthritis may be triggered by diuretics, which interact with tubular excretion of urate.

### 1.7 Cholelithiasis

Patients with cyanotic CHD have an increased turnover of heme due to erythrocytosis, which results in an increased concentration of unconjungated bilirubin in the bile. Thus, Eisenmenger patients are at risk for calcium bilirubinate gallstones complicated by acute cholecystitis. This serious, life-threatening complication can be further complicated by bacteremia (gram-negative sepsis) and endocarditis. 

Patients with an acute cholecystitis must be referred to a tertiary care center where a multidisciplinary team with expertise in the management of these patients can provide the best care. An endoscopic retrograde cholangiography and papillotomy are attractive in the presence of a retained stone in the common bile duct to avoid general anesthesia and laparotomy with all their implications. However, care support by an anesthetist with special expertise in the management of patients with PAH is advisable during this procedure. Cholecystectomy must be considered and is best managed by a multidisciplinary team in a tertiary center with an established adult CHD program.

### 1.8 Hypertrophic Osteoarthropathy (HOA)

Patients with the ES often suffer from arthralgias, typically from of the knees and ankles. These arthralgias are often a manifestation of hypertrophic osteoarthropathy.

HOA is characterized by local cell proliferation and new osseous formation and periostitis, occuring particularly in the metacarpal, metatarsal, and long bones of the forearms and legs [42]. The exact etiology of HOA is unknown. However, HOA is often associated with right-to-left shunt lesions bypassing the pulmonary circulation, or with an intrapulmonary shunting of blood. Presumably, factors or mediators in the systemic venous circulation that normally are removed or inactivated in the lung, are causative for HOA, as they escape an inactivation in the pulmonary capillary bed due to the right-to-left shunt. Due to the right-to-left shunt, megakaryocytes can reach the bones *via* the systemic circulation without fragmentation in the pulmonary microvasculature. They may activate local endothelial cells through the release of platelet-derived growth factor, initiating clubbing. For treatment Salsalate, an nonacetylated analog of aspirin, or oral corticosteroids may be used, while nonsteroidal anti-inflammatory agents should be avoided. 

### 1.9 Travel to High Altitude / Air Flight

Traveling is an important life quality factor for many patients. However, the safety of traveling in a commercial aircraft was questioned for Eisenmenger patients who were frequently restricted from traveling in the past. A decrease in oxygen saturation because of the reduced cabin pressure was the major concern in these fragile patients who were advised to take supplementary oxygen or even to avoid air travels. 

Aircrafts are pressurized to a cabin pressure altitude corresponding to a pressure at 1800 and 2400 m (6000 to 8000 feet) above sea level. A Dutch group designed a unique study including cyanotic patients and healthy individuals and studied both groups during simulated and actual flights [44]. Commercial air travel was well tolerated. Actual decrease in oxygen saturation during ascent followed a similar pattern in patients and healthy controls and no patient experienced an adverse effects. A recently published retrospective study confirmed good tolerance of traveling in a commercial aircraft and experiences of the Dutch study [45]. Patients with ES do not need to be advised against air travels and do not require in-flight nasal oxygen. 

#### Risk Reduction Strategie

- Avoidance of travel and non-travel related stress (flight organization well in advance, easy transportation, avoidance of flying at busy periods; availability of luggage carrier, wheelchair, etc.);
- Avoidance of volume depletion (low humidity in commercial aircrafts!); avoidance of alcoholic drinks;
- Prevention of deep vein thrombosis with the potential of paradoxical emboli (business class travel, aisle seat, extension of the legs, periodic walks, fluid intake).

**Exposure to high altitude:** Exposure to high altitude (>1500 m above sea level) may be safe. Indeed, Eisenmenger patients traveled from Europe to Grand Canyon without adverse effects. However, a gradual ascent on land is important and a time for acclimatization may be wise. In addition, a cable car or another transport medium should be available for an immediate descent in the case of health problems and an emergency. Exercise at high altitude must be very limited and strongly restricted by symptoms.

### 1.10 Infective Endocarditis

Patients with ES carry a high risk for endocarditis with high morbidity and mortality. They require meticulous endocarditis prophylaxis [46, 47]. The importance of excellent oral hygiene must be emphasized to prevent endocarditis.

### 1.11 Pregnancy and Contraception

In our view, ES is even today an absolute contraindication to pregnancy. In women with ES pregnancy is associated with a substantial risk for fetus and mother: as the systemic vascular resistance drops during pregnancy, the right-to-left shunt increases and the arterial oxygen saturation decreases. At particular risk are those patients who are already in a functional class III or IV. According to classic data spontaneous abortion occurs in up to 40%, premature delivery in 50%, and term delivery in only 25% of pregnancies [48, 49]. Moreover, 30% of the infants suffer from intrauterine growth retardation and the perinatal mortality is also high (8% to 28%) [48, 49].

Maternal mortality ranges from 30% to 60%, and may be attributed to syncope, thromboembolism, hypovolemia, hemoptysis or preeclampsia [48, 49]. Most deaths occur either during delivery or within the first weeks after. 

Although therapeutic abortion bears a risk, termination of pregnancy should be recommended because of the considerable maternal risk. Woman declining abortion need a multidisciplinary approach, including at least experienced congenital cardiologists, obstetricians, anesthetists, and neonatologists. In selected cases, nebulized Iloprost or intravenous prostaglandin therapy may be indicated, even despite the lack of evidence and the unknown effects on the fetus [50].

Pregnant women with ES should be hospitalized after the 20^th^ week of pregnancy - or earlier if clinical deterioration occurs. 

In case of dyspnea, supplemental oxygen should be administered, which may decrease the degree of PAH and right-to-left shunting to some degree. Congestive heart failure should be treated with digoxin and diuretics. The woman's fluid status, systemic arterial pressure and oxygen saturation should carefully be assessed. 

The best mode of delivery is still a matter of discussion, and either vaginal delivery (with keeping the second stage of labor short by elective use of vacuum-assist delivery or forceps delivery) or elective cesarean section are possible [50]. 

In any case, significant blood loss may induce arterial hypotension and increase the amount of the right-to-left shunt and cyanosis. Arterial hypotension should be treated immediately with parenteral volume replacement and vasopressors.

Although no evidence based data exist, some centers use low-molecular weight heparin for thromboprophylaxis from the 20^th^ week of pregnancy. Prior to delivery, intravenous heparin (aPTT-controlled) can be started instead, as subcutaneous heparin can cause a persisting anticoagulatory effect. Low-molecular weight heparin can be reinstituted usually 1 or 2 days after delivery, if the patient is stable. When heparin is administered too early after delivery, excessive bleeding, leading to maternal death, may occur. 

Prevention of pregnancy and counseling about contraceptives is of outstanding importance. Best suitable contraception are the progestogen-only contraceptives without thrombophilic properties (pills, intrauterine devices, subdermal implantats), including progestogen-only pills (e.g. Cerazette^®^), Mirena^®^ as an intrauterine system, Implanon^®^ as a subdermal implant or the subdermal injection of a progestogen [51, 52]. 

As the ET-blocker Bosentan may reduce the efficacy of orally given progestogens due to enzyme induction, individualized recommendations from an expert are mandatory in these special situations. The use of an intrauterine progestogen coated coil might be a safe and efficient option, particularly, as the risk of a local infection and a consecutive endocarditis seem to be low. 

Combined oral contraceptives are contraindicated because of the increased risk for thromboembolic complications associated with their use. Barrier methods, such as condoms and diaphragms, have an unacceptably high failure rate. Tubal ligation is an effective method of contraception, however, carrying a significant procedural risk itself. 

### 1.12 Non-Cardiac Surgery

Patients with ES are vulnerable to any hemodynamic changes and are at risk for any non-cardiac surgical procedure. The increased pulmonary vascular resistance (Eisenmenger physiology) precludes rapid adaptive mechanisms to any hemodynamic change caused by anesthetics, fluid shifts and/ or surgery [53, 54]. Every surgical procedure carries a high risk of morbidity and a substantial risk of mortality. No prospective study evaluated the risk of non-cardiac surgery in this population.

The perioperative risks include:

- Increase in right-to-left shunt and increased cyanosis secondary to decrease in systemic vascular resistance leading to collapse and death;
- Depression of ventricular function due to a sudden increase in systemic vascular resistance;
- Arrhythmias (supraventricular/ ventricular);
- Risk of bleeding (hemostatic abnormalities);
- Thromboembolic complications (thrombotic diathesis).

**Risk reduction strategies** to reduce the perioperative risk [8]:

- Meticulous preoperative evaluation (medical history, physical examination, ECG, chest x-ray, complete blood count, blood chemistry, coagulation parameters, Doppler echocardiography);
- Provision of care by an expert team including cardiac anesthetist with expertise in the management of Eisenmenger patients;
- Local anesthesia whenever possible;
- The choice of general anesthesia versus epidural-spinal anesthesia is controversial. General anesthesia is preferable. Regional anesthesia may be hazardous as it results in sympathetic blockade and decrease in both afterload and preload; a bleeding diathesis is a contraindication to epidural/ spinal anesthesia;
- Preoperative phlebotomy can be considered if the hematocrit exceeds 65%. This strategy may increase platelet count and reduce the risk of intraoperative bleeding. The blood withdrawn should be reserved for autologous blood donation if required [53, 54];
- Careful intraoperative monitoring to detect sudden pressure and volume changes, and pulse oximetry to assess oxygen saturation (changes in vascular resistance alters right-to-left shunting);
- Surgery must be performed by an experienced surgeon; even a so-called simple surgical procedure (e.g. appendectomy) is not simple, it is demanding for the entire team;
- Endocarditis prophylaxis and
- use of an air filter.

Eisenmenger patients are at risk for death even after the acute phase of the procedure; this implies that these patients need postoperative surveillance and should not be discharged too early.

## 2 SURGICAL ASPECTS

### 2.1 Pulmonary Artery Banding

Pulmonary artery banding for patients with Eisenmenger physiology was a matter of big debates in the late 1990s. Batista reported about a 19 year female with pulmonary artery pressure at systemic level and grade IV pulmonary vascular changes due to an atrial (ASD) and ventricular septal defect (VSD) [55]. Although there was increased right-to-left shunting, worsening of cyanosis was only transient after pulmonary artery banding. Cyanosis improved gradually, the patient was getting pink at rest and during exercise and underwent successful closure of her ASD and VSD. The mechanism of this pulmonary vascular remodeling remains unclear. There is the hypothesis that decreased oxygen saturation and lowered pulmonary artery pressure might have induced apoptosis and remodeling of the pulmonary vascular bed. Batista [55] performed the same surgical approach in six other patients with similar clinical response. This new concept, however, has remained experimental and is not performed anymore.

### 2.2 Transplantation

Transplantation is a final therapeutic option for patients with poor prognosis and quality of life. However, timing and appropriateness of transplantation remain difficult decisions in patients with ES. The following challenges have to be addressed before listing for transplantation:

1. Timing of listing is difficult because survival of Eisenmenger patients is hard to predict and risk stratification scores established for patients with end-stage acquired heart disease do not apply to these patients. Survival of patients with ES is significantly better than in those with other forms of pulmonary arterial hypertension and their survival pattern cannot be compared between these two groups [30, 38, 56]. Median survival was 52.6 years in a population of 109 patients followed during 6.3 years [38]. Predictors of survival include: complexity of the underlying congenital heart defect, age at symptoms or referral to a tertiary care center, right heart failure symptoms, syncope, creatinine, atrial arrhythmias, function class, increased precordial ECG voltage as an index of RV hypertrophy, uric acid, high right atrial pressure [1, 30, 38, 43, 57].
2. Eisenmenger patients have adapted to their limited working capacity since childhood and present often late to the transplant team.
3. Technical considerations are significant: complexity of the underlying anatomy, previous sternotomies and thoracotomies, major aorto-pulmonary or pleuro-pulmonary collaterals, risk of bleeding. Previous operative procedures seem to have a negative impact on survival after transplantation and the existence of extensive pleuro-pulmonary collateral vessels are considered a contraindication for heart-lung transplantation [58].
4. Heart-lung transplantation or bilateral lung transplantation/ single lung transplantation and repair of the congenital heart defect to increase the donor pool in the era of organ shortage? Single/ bilateral lung transplantation and repair of the congenital heart defect are attractive and may be feasible in patients with isolated defects (e.g. PDA, ASD, VSD). Bilateral lung transplantation and cardiac repair is a feasible option and can be performed as an alternative procedure to heart-lung transplantation in Eisenmenger patients without an increase in early or medium-term morbidity and mortality and results were comparable with heart-lung transplantation or with lung transplantations performed for other indications [59, 60]. However, another study showed a highly significant benefit of heart-lung transplantation over lung transplantation for patients with a VSD [61]. This is also consistent with another single center experience [58].
5. The shortage of donor organs does have implications on the wait times for patients with CHD, which is longer than for other diagnostic groups. In addition, heart-lung transplantation is less attractive for transplant teams as the donor heart cannot be preserved for another recipient.

Transplantation in patients with ES must be performed in a tertiary care center with an established close collaboration between the transplant team and the CHD specialists (multidisciplinary team approach). Potential candidates for transplantation must be referred early to the transplant team as the question when to list an individual patient is hard to answer and the decision has to be made individually for every patient based on the medical history and the clinical presentation. The type of operation -heart-lung transplantation or double lung transplantation plus repair of the cardiac defect- depends on underlying CHD, co-morbidities and on the experiences of the transplant team.

Heart-lung transplantation can be a successful procedure for Eisenmenger patients with end-stage disease if the procedure is performed by an experienced team [58, 61, 62]. One, 5-year and 10-year survival ranges between 70% and 80%, 50% and 70%, 30% and 50%, respectively [58, 61, 62]. Long-term complications and cause deaths include chronic rejection despite improved immunosuppressive therapy and infectious disease.

Transplantation remains a therapeutic option for selected patients and successful transplantation improves quality of life in severely disabled patients. However, patient selection and timing of listing for transplantation are critical and challenging, because Eisenmenger patients have very long natural survival patterns compared to patients with other forms of pulmonary hypertension. Potential candidates must be referred to a dedicated team sooner rather than later.

## 3 LABORATORY PRECAUTIONS

Table **3** summarizes routine laboratory assessment of patients with cyanotic congenital heart disease (Table **3**). Caution is required for accurate measurement of coagulation parameters, hematocrit and blood glucose.

### 3.1 Coagulation Parameters

Frequently, physicians do not appreciate inaccurate measurements of coagulation parameters if blood is withdrawn into regular tubes with a standard amount of citrate anticoagulant. Plasma volume is decreased in Eisenmenger patients due to secondary erythrocytosis and elevated hematocrit. Thus, the amount of citrate anticoagulant is excessive for patients with cyanosis and secondary erythrocytosis. Accurate measurement of coagulation factors requires adjustment of the amount of liquid anticoagulants according to the hematocrit level when hematocrit is higher than 55%.

### 3.2 Hematocrit

Microhematocrit centrifugation results in plasma trapping and falsely raised hematocrit. Thus, determination of the hematocrit level must base on automated electronic particle counts.

### 3.3 Blood Glucose

Reduced blood glucose levels are not uncommon in cyanotic patients because of the increased *in vitro* glycolysis, which results from the increased number of red blood cells. Sodium fluoride must be added to the tube to prevent red cell glycolysis and artificial ‘hypoglycemia’.

## Figures and Tables

**Fig. (1):**
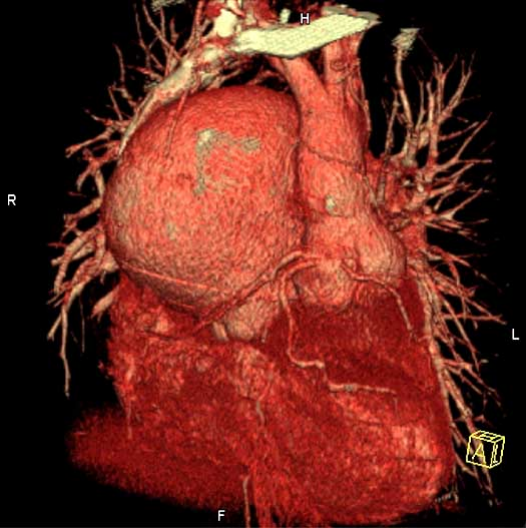
Aneurysm of the pulmonary trunk with a deviation of the ascending aorta.

**Table 1:** Clinical Assessment of Patients with Cyanotic Congenital Heart Disease

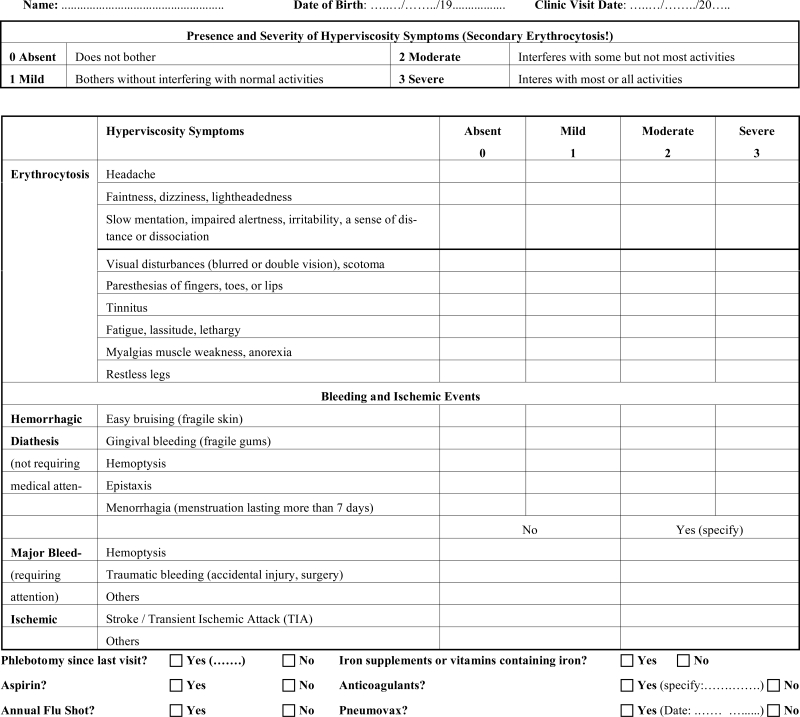

**Table 2:** Management of Hyperviscosity Symptoms

Volume depletion → Volume replacement; Iron deficiency (anemia) → Iron replacement; Moderate to severe symptoms due to secondary erythrocytosis → phlebotomy and withdrawal of 300-500 cc of whole blood.

**Table 3:** Routine Laboratory Assessment

1) Hematology
Hemoglobin, hematocrit, white blood cell, platelets, red cell indices (MCV, MCHC);
2) Iron stores
Ferritin, transferrin, transferrin saturation;
3) Vitamines
Vitamine B12, folic acid in the presence of iron deficiency and normal or elevated MCV;
4) Biochemistry
Creatinine, electrolytes, uric acid;
5) Neurohormones
BNP or NT-pro-BNP;

**Table 4:** Risk Reduction Strategies

Avoidance of volume depletion; Avoidance of iron deficiency (no routine phlebotomies); Avoidance of cigarette smoking and recreational drug use; Precaution or avoidance of drugs that impair renal function; Use of an air filter for all intravenous lines to avoid air embolism; Avoidance of infectious disease: Annual flu shot, pneumovax 23, every 5 years; Prompt therapy of respiratory tract infections; Avoidance of strenuous exercise/ stress (traveling).

## Footnotes

1 Braun S, Mebus S, Eicken A, Hager A, Hess J, Kaemmerer H. Coagulation parameter and platelet function in whole blood samples of adults with cyanotic congenital cardiac disease. Cardiology in the Young 2008; 18 (Suppl. 1):76
